# Roles of the lipopolysaccharide biosynthesis-related gene *HP0858* in the fitness of *Helicobacter pylori* and its virulence in *Galleria mellonella*

**DOI:** 10.1080/21505594.2025.2548620

**Published:** 2025-08-24

**Authors:** Tram Thi Hong Nguyen, Pei-Chun Chen, Po-Chuan Wang, Wen-Han Wang, Chung-Yu Lan, Steffen Backert, Mou-Chieh Kao

**Affiliations:** a Institute of Molecular Medicine, National Tsing Hua University, Hsinchu, Taiwan; b Department of Gastroenterology, Hsinchu MacKay Memorial Hospital, Hsinchu, Taiwan; c Institute of Molecular and Cellular Biology, National Tsing Hua University, Hsinchu, Taiwan; d Department of Life Science, National Tsing Hua University, Hsinchu, Taiwan; e School of Medicine, National Tsing Hua University, Hsinchu, Taiwan; f Division of Microbiology, Department of Biology, Friedrich-Alexander-Universität Erlangen-Nürnberg, Erlangen, Germany

**Keywords:** *Helicobacter pylori*, lipopolysaccharide, ADP-heptose, bi-functional kinase/adenylyltransferase (RfaE/hlde/hp0858), protein glycosylation, *Galleria mellonella*

## Abstract

*Helicobacter pylori* is a pathogenic bacterium associated with the development of gastric cancer and other gastric disorders. One of its major virulence factors, lipopolysaccharide (LPS), plays a crucial role in maintaining bacterial integrity, mediating host adhesion, and modulating the immune response. Recent studies have indicated that ADP-heptose, an intermediate in the heptose biosynthetic pathway involved in the LPS synthesis cascade, is a novel pathogen-associated molecular pattern for *H. pylori*. This study focuses on the *HP0858* gene, which is predicted to encode RfaE/HldE, an enzyme with kinase and ADP-transferase activities essential for heptose production. An *HP0858* gene-disrupted mutant was first generated, and the resulting mutant exhibited a truncated LPS structure, confirming its role in LPS biosynthesis. The *HP0858*-deficient mutant displayed increased sensitivity to the detergent SDS and the antibiotic novobiocin, heightened surface hydrophobicity, and a propensity for autoaggregation. Additionally, the mutant exhibited reduced adhesion and internalization capabilities, a diminished elongation phenotype, and failed to induce IL-8 secretion in infected gastric AGS cells. In an *in vivo Galleria mellonella* infection model, the *HP0858* knockout mutant showed significantly attenuated virulence, as no bacterial load was detectable in the larvae’s hemolymph 48 h post-infection, unlike the wild-type strain. Finally, we provided evidence that the enzyme encoded by *HP0858* is involved in a general protein glycosylation system linked to LPS biosynthesis, specifically glycosylating the adhesin AlpA. These findings highlight the essential role of RfaE/HldE/HP0858 in LPS biosynthesis and bacterial virulence, making it a promising target for future therapeutic interventions against *H. pylori* infections.

## Introduction

*Helicobacter pylori* (*H. pylori*) infects almost half of the world’s population and is identified as a common cause of gastritis and gastric and duodenal ulcers. Although most infected people have no clinical symptoms, *H. pylori* indeed causes inflammation in host cells [1], and the infected individuals may further develop gastric cancers or even mucosa-associated lymphoid tissue (MALT) lymphoma [2,3].

The adherence of bacteria to host cells is an essential step for *H. pylori* colonization, which helps bacteria fight against peristalsis, acquire nutrients, and deliver bacterial effector molecules to host cells. One group of bacterial virulence factors contains the bacterial outer membrane proteins (OMPs) recognized as adhesins, which contribute to the colonization of human gastric mucosa and the pathogenesis of *H. pylori* infection. Among these adhesins that are reported for binding *H. pylori* to the gastric epithelial cells, blood-group-antigen-binding adhesin A (BabA), sialic-acid binding adhesin A (SabA), outer inflammatory protein A (OipA), adherence-associated lipoproteins A and B (AlpA/AlpB), and *Helicobacter* outer membrane protein Q (HopQ) are the most studied OMPs in establishing a bacterial role in colonization [4].

**Figure 1.**
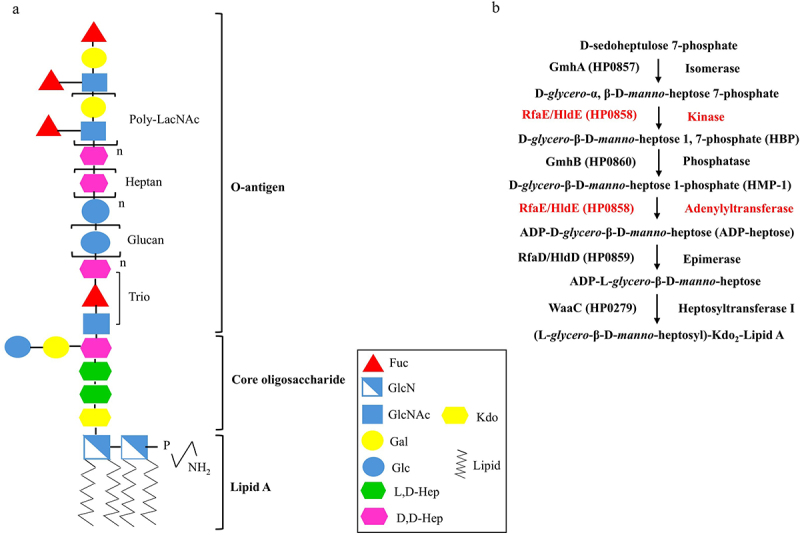
Diagram of the LPS structure and heptose biosynthetic pathway in *H. pylori*. (a) The redefined LPS structure of *H. pylori* strains 26,695 and G27. (b) The heptose biosynthetic pathway in *H. pylori* contains five steps. It is catalyzed sequentially by GmhA (sedoheptulose 7-phosphate isomerase), RfaE/HldE (bifunctional D-β-D-heptose 7-phosphate kinase/D-β-D-heptose 1-phosphate adenyltransferase), GmhB (D-α, β-D-heptose 1,7-bisphosphate phosphatase), RfaE/HldE and RfaD/HldD (ADP-D-β-D-heptose epimerase). The resulting ADP-LD-Hep is then transferred to the core region of LPS by WaaC (heptosyltransferase I). The enzyme tested in this study is marked in red color.

Lipopolysaccharide (LPS) is a crucial surface determinant for *H. pylori* colonization and persistence in the gastric niche. LPS is a large and complex glycolipid localized in the outer membrane of Gram-negative bacteria. It has many functions, such as mediating the interaction between bacteria and their surrounding environment and maintaining the barrier feature of bacterial outer membranes. A recent analysis has redefined the *H. pylori* LPS structure, which suggested that LPS in *H. pylori* is composed of lipid A, a short and conserved core oligosaccharide, and a longer O-antigen embracing a domain previously assigned as the outer core region (Figure 1(a)) [5]. Compared to other *Enterobacteriaceae* such as *Escherichia coli*, *Neisseria meningitides*, and *Pseudomonas aeruginosa* [6,7], the constitutive modifications of *H. pylori* lipid A result in weak activation of toll-like receptor 4 (TLR4), thus evading host innate immune response and promoting chronic infection [8]. The latest redefined core oligosaccharide of *H. pylori* LPS is known as a conserved region in most Gram-negative bacteria. It contains one 3-*deoxy*-D-*manno*-oct-2-ulsonic acid (Kdo), two LD-heptose residues (heptose I and heptose II), and one DD-heptose (heptose III) with a branched disaccharide composed of galactose and glucose. This part of LPS can contribute to *H. pylori* colonization and pathogenesis. The outermost domain of *H. pylori* LPS is O-antigen, which has a unique structure consisting of a conserved trisaccharide (-GlcNAc-Fuc-DD-Hep-), homopolymers named DD-heptane and α-1, 6-glucan, and variable Lewis antigens which can mimic the Lewis antigens in the human stomach and evade the host’s immune recognition [9–12].

Heptose is one of the main components of the LPS core region. As early as 2001, Fischer *et al*. proposed that an unknown factor could be injected into the host cell via the type IV secretion system (T4SS) during *H. pylori* infection, and thus trigger cytokine interleukin-8 (IL-8) activation in host cells [13]. CagA was later identified as one of the injected T4SS factors and a potent contributor to IL8 activation in many strains [14]. In 2015, D-*glycero*-β-D-*manno*-heptose 1, 7-bisphosphate (HBP), an intermediate metabolite of the heptose biosynthetic pathway, was identified as another potent effector of IL-8 activation by *Neisseria* species [15]. More recently, it was reported that proteins α-kinase 1 (ALPK1) and TRAF-interacting protein with forkhead-associated domain (TIFA) are critical components in the response to HBP during *H. pylori* infection, leading to NF-κB activation and IL-8 transcription [16–18]. However, the latest research indicated that this immune-regulatory pathway is mainly elicited by ADP-heptose, another intermediate metabolite of the heptose biosynthetic pathway, rather than HBP [19]. The complete ADP-LD-heptose biosynthetic pathway in *H. pylori* contains five steps that are similar to the corresponding biosynthetic pathway present in most Gram-negative bacteria (Figure 1(b)) [20,21]: (1) the starting D-sedoheptulose 7-phosphate is first converted into D-*glycero*-α, β-D-*manno*-heptose 7-phosphate by an isomerase GmhA (encoded by *HP0857* in *H. pylori*); (2) the resulting product is phosphorylated to form D-*glycero*-β-D-*manno*-heptose 1, 7-bisphosphate (HBP) by the kinase activity of a bi-functional enzyme, RfaE/HldE (encoded by *HP0858* in *H. pylori*); (3) HBP is then dephosphorylated to form D-*glycero*-β-D-*manno*-heptose 1-phosphate (HMP-1) by a phosphatase GmhB (encoded by *HP0860* in *H. pylori*); (4) through nucleotide activation by the adenylyltransferase activity of RfaE/HldE, ADP-D-*glycero*-β-D-*manno*-heptose (ADP-DD-Hep) is formed; (5) ADP-DD-Hep is epimerized to ADP-L-*glycero*-β-D-manno-heptose (ADP-LD-Hep) by the epimerase RfaD/HldD (encoded by *HP0859* in *H. pylori*). The resulting ADP-LD-Hep is then transferred to Kdo in the LPS core region by the heptosyltransferase I WaaC (encoded by *HP0279* in *H. pylori*).

*Galleria mellonella* (greater wax moth) is an efficient invertebrate model for studying bacterial virulence [22]. Compared to the mammalian models of bacterial infection, *G. mellonella* larvae offer several advantages, including no requirement for time-consuming ethical certification, being convenient to use and handle, generating results quickly, and having a low experimental cost. In particular, *G. mellonella* larvae can be injected with precise doses of bacterial concentration and incubated at 37°C, corresponding to the host body temperature. More importantly, *G. mellonella* larvae have an innate immune system with phagocytic cells known as hemocytes, which can engulf and kill invading pathogens. *G. mellonella* has been used as an *in vivo* model over recent years to study the virulence of various *Helicobacter* species by determining the survival rate of larvae and viable bacteria in the hemolymph of larvae during a time course of infection [23,24].

In previous studies, we investigated the functions/structures of HP0857 (GmhA), HP0860 (GmhB), and HP0859 (RfaD/HldD) proteins involved in the heptose biosynthetic pathway. We demonstrated that those proteins play crucial roles in LPS core biosynthesis and significantly influence bacterial fitness and virulence [25–27]. To clarify the remaining putative gene involved in heptose biosynthesis, we aimed to explore the involvement of HP0858 (RfaE/HldE) protein in LPS biosynthesis, bacterial physiology, as well as its virulence assessment in an *in vivo* model newly launched in this study. A knockout mutant and its corresponding complemented strain were constructed, and the effects of the *HP0858* mutations on LPS profile, pathogenesis, and bacterial virulence assessment in *G. mellonella* larvae were examined. Together, the experimental results from this study shed new light on the function of *H. pylori* LPS genes, which in turn can serve as promising novel targets for anti-microbial therapy in the future.

## Materials and methods

### Bacterial strains, plasmids, and growth conditions

Bacterial strains and plasmids used in this study are listed in Table 1. Unless stated otherwise, stationary-phase *H. pylori* cultures were used throughout the assays. This growth phase represents a state of nutrient limitation and environmental stress, conditions that more accurately simulate the physiological challenges *H. pylori* faces within the gastric mucosa. Therefore, employing stationary-phase bacteria offers a more representative model for investigating bacterial adherence and other host-pathogen interaction phenotypes examined in this study. *H. pylori* strain 26,695 [KE26695] (ATCC 700,392; CagA^+^, VacA^+^) and its derived mutants were inoculated on sheep blood (5%, w/v) agar plates and incubated for 48–72 h at 37°C under a microaerophilic environment (5% O_2_, 10% CO_2_, and 85% N_2_). Bacteria were scraped from the agar plates and resuspended in flasks containing Brucella broth media (BD Biosciences, Franklin Lakes, NJ, USA) with 10% fetal bovine serum (FBS, Biological Industries, Kibbutz Beit Haemek, Israel) and 1% Iso Vitalex (Taiwan Prepared Media, Taipei, Taiwan). Flasks were then placed into an anaerobic chamber and cultivated for 48 h under microaerophilic conditions at 37°C with shaking at 140 r.p.m.

**Table 1.** Bacterial strains and plasmids used in this study.

Strain/Plasmid		Characteristic and marker^1^	Source
	*E. coli* DH5α	The host for the construction of pGEM-T-HP0858:Cm^r^ clone	Invitrogen, Carlsbad, CA, USA
Strain	*H. pylori* 26,695	*H. pylori* whole-genome sequencing strain, isolated from the stomach of a patient with gastritis	ATCC^2^ 700392
	KO 0858	*H. pylori* 26,695 strain with a chloramphenicol resistance cassette in *HP0858*; Cm^r^	This study
	Com 0858	KO 0858 with *HP0858* insertion in *HP0954* (RdxA); Met^r^, Cm^r^	This study
	pGEM-T	T-A cloning vector; Amp^r^	Promega, Madison, WI, USA
	pJET1.2/blunt	Blunt PCR cloning vector	Thermo Fisher Scientific
	pGEM-T-HP0858	pGEM-T with *HP0858* DNA fragment; Amp^r^	This study
Plasmid	pGEM-T- HP0858:Cm^r^	pGEM-T with *HP0858* interrupted with a chloramphenicol resistance cassette; Cm^r^	This^2^ study
	pGEM-T-RdxA_L_- P_HP1563_-HP0858-T7_ter_ -RdxA_R_	pGEM-T with *HP0858* insertion in *HP0954* (RdxA); Met^r^, Cm^r^	This study

^1^Amp^r^, ampicillin-resistant; Cm^r^, chloramphenicol-resistant; and Met^r^, metronidazole-resistant.

^2^ATCC: American Type Culture Collection.

### Construction of the *HP0858* knockout mutant and the corresponding complemented strain

The *HP0858* knockout mutant was constructed using a strategy based on gene splicing by overlapping extension (SOEing) [28–30]. For the construction of the gene disruption mutant, the upstream (457 base pairs (bps)) and downstream (463 bps) regions of the *HP0858* gene were amplified by polymerase chain reaction (PCR) using *Taq* DNA polymerase (Mdbio, Taipei, Taiwan) with the genomic DNA extracted from *H. pylori* 26,695 wild-type (WT) strain using the primer pairs KO0858F1-KO0858R1/KO0858F2-KO0858R2 (Table 2). The above two sets of generated PCR products were then joined via SOEing PCR with the primer pair KO0858F1-KO0858R2 carrying a *BamHI* restriction site in the PCR products for subsequent insertion of an antibiotic resistance cassette to disrupt the *HP0858* gene. The amplified 920-bp fragment of *HP0858* was purified and ligated into the pGEM-T Easy Vector (Promega, Madison, WI, USA) with T4 DNA ligase (New England Biolabs, Ipswich, MA, USA) to generate the pGEM-T-HP0858 plasmid. After ligation, the plasmid containing the target fragment was inserted with a chloramphenicol resistance gene at the *BamHI* restriction site and later transformed into *E. coli* strain DH5α (Thermo Fisher Scientific, Waltham, MA, USA). The resultant plasmid DNA was isolated and used for natural transformation into *H. pylori* 26,695 WT strain. The gene disruption mutant was selected using chloramphenicol-containing agar plates.

**Table 2.** Sequences and restriction sites of the primers used in this study.

Primer	Restriction enzyme site	Sequence (5’→ 3’)^3^
KO0858F1^1^ KO0858R1^1^ KO0858F2^1^ KO0858R2^1^ Com0858F1^2^ Com0858R1^2^ Com0858F2^2^ Com0858R2^2^	- *BamHI* *BamHI - NcoI - - KpnI*	TCTTAGTCATAGGCGATCTG GAGTTCAAAGGATCCAACAC GTGTTGGATCCTTTGAACTC TCTTCTAAACTCGCTAACGC^2^ AGTACCATGGTTGACTTGGATTTC^3^ CGCCTATGACTAAGATTTTTTTCATATCGTAACTCC GGAGTTACGATATGAAAAAAATCTTAGTCATAGGCG AGTAGGTACCTCAATCATTGC

^1^Primers used in the construction of the *HP0858* knockout mutant.

^2^Primers used in the construction of the HP0858 complemented strain.

^3^The sequences of restriction enzyme sites are underlined.

Gene complementation was conducted to complement the HP0858 knockout mutation by inserting the WT gene into the locus of the *rdxA* (*HP0954*) gene. A 1736-bp fragment containing the promoter region of the *HP1563* gene (350 bps) and the open reading frame (ORF) of the *HP0858* gene (1386 bps) was amplified by PCR using *Pfu* DNA polymerase (Promega) from *H. pylori* 26,695 genomic DNA as the template using the primer pairs Com0858F1-Com0858R1 and Com0858F2-Com0858R2, respectively (Table 2). The obtained fragments were fused together after overlapping extension PCR with the primer pair Com0858F1-Com0858R2. This extension PCR product was next ligated into the pJET1.2/blunt Cloning Vector (Thermo Fisher Scientific) and transformed into *E. coli* strain DH5α. After purification, the isolated plasmid was digested with restriction enzymes *NcoI* and *KpnI*, resulting in an insert containing the promoter region of the *HP1563* gene and the *HP0858* gene. The resultant fragment was then ligated into the pGEM-T-RdxA_L_-T7_ter_-RdxA_R_ plasmid, which had been *NcoI*/*KpnI*-digested to create the pGEM-T-RdxA_L_-P_*HP1563*_-*HP0858*-T7_ter_-RdxA_R_ plasmid. After isolation, the consequent plasmid DNA was used for natural transformation into the *HP0858* disruption mutant, and the corresponding complemented strain was selected using chloramphenicol- and metronidazole-containing agar plates. Finally, the *HP0858* knockout mutation and its corresponding complemented mutation were confirmed by PCR amplification using primers spanning DNA upstream and downstream of the target sites, which was then confirmed through sequencing by Mission Biotech (Taipei, Taiwan). The plasmid DNA extraction and gel extraction kits were bought from Biokit (Miaoli, Taiwan). The restriction enzymes were purchased from New England Biolabs. The antibiotics used to select mutants were bought from Sigma-Aldrich (St. Louis, MO, USA).

### Bioinformatics analysis

The sequences of the *HP0858* gene and its corresponding protein were searched on the Kyoto Encyclopedia of Genes and Genomes (KEGG) website (https://www.genome.jp/kegg/) and UniProt (https://www.uniprot.org/), respectively. The conserved domain searching of the target protein was conducted by using the Conserved Domains Database (CDD) on the National Center for Biotechnology Information (NCBI) website (https://www.ncbi.nlm.nih.gov/Structure/cdd/cdd.shtml). The protein sequence alignment with various bacterial species was generated by Clustal Omega (https://www.ebi.ac.uk/Tools/msa/clustalo/) and colored by Jalview (https://www.jalview.org/).

### LPS profile analysis

Bacteria were first scraped from agar plates and cultivated under liquid culture conditions for 48 h. After harvesting by centrifugation (4,000* × g*, 10 min, 4°C) to collect the bacteria, the obtained pellets were resuspended in 600 μL of the lysis buffer (1 M Tris-HCl, 1 M NaCl, 0.5 M EDTA, 10% SDS). The BCA protein assay kit was used to evaluate the amount of protein in the sample. The whole-cell lysate from the sample was added with 15 μL of proteinase K (20 mg/mL, Sigma-Aldrich), followed by the addition of deionized water to a total volume of 150 μL, and the resulting suspension was incubated at 60°C for 120 min. Then, 150 μL of 90% hot phenol solution (70°C) was added to the suspension, and the resulting mixture was incubated in a 70°C water bath for 15 min with gentle inversion every 5 min. The mixture was then cooled down on ice for 10 min. Finally, the upper aqueous phase containing LPS was collected by centrifugation (10,000*×*g, 10 min, 4°C). LPS profiles were analyzed by silver staining. The LPS samples were separated by 15% Tricine gel and fixed in 150 mL fixing solution (25% isopropanol and 7% acetic acid) overnight. The obtained gel was then soaked in the oxidizing solution (27% ethanol, 0.7% periodic acid, 0.3% acetic acid) for 10 min and washed for 30 min with distilled water three times. After oxidation, the gel was transferred into the staining solution (18.7% 1N NaOH, 1.3% ammonium hydroxide, 0.6% silver nitrate), stained for 25 min, and washed for 10 min with distilled water three times. Finally, the gel was developed by a reduction reaction in the developing solution (0.074% formaldehyde and 0.05% citric acid). The purified LPS samples were also separated by 15% sodium dodecyl sulfate polyacrylamide gel electrophoresis (SDS-PAGE), transferred to 0.45 µm nitrocellulose membranes (NC membrane, Millipore, Billerica, MA, USA) for immunoblotting analysis. The transferred membranes were blocked with 5% skim milk in phosphate-buffered saline (PBS) for 1 h at room temperature, followed by incubation overnight at 4°C with the primary monoclonal mouse-anti-Lewis X antibody and mouse-anti-Lewis Y antibody (1:1,000, both from Abcam, Cambridge, UK), respectively. Afterward, the membranes were washed and incubated with the secondary HRP goat anti-mouse IgM antibody (1:10,000, Thermo Fisher Scientific) for 1 h at room temperature. After washing, the membranes were visualized by the Bio-Rad ChemiDoc MP Imaging System (Hercules, California, USA).

### Subcellular fractionation and immunoblotting analysis

*H. pylori* was cultured to the stationary phase for 48 h and then harvested by centrifugation (4,000×g, 10 min, 4°C) to collect the bacterial pellet. After washing once with 1×PBS and centrifugation at 4,000×g for 10 min, the obtained bacteria were resuspended in 20 mM Tris-HCl (pH 7.4) and broken by sonication (30% amplitude for 10 min with a 5 s interval in each cycle). The debris was first removed by centrifugation (10,000×g, 10 min, 4°C), and the whole cell lysate was further fractionated by ultracentrifugation. After the first ultracentrifugation (50,000*×*g, 45 min, 4°C), the supernatant was considered the cytoplasm/periplasm (C/P) fraction, and the proteins inside were collected by acetone precipitation. The obtained pellet, considered the total membrane fraction, was resuspended in 20 mM Tris-HCl (pH 7.4) supplemented with 2% N-lauroylsarcosine sodium (Sigma-Aldrich), followed by a second ultracentrifugation at 50,000* × g* (45 min, 4°C). Finally, the inner membrane (IM) fraction contained in the obtained supernatant was collected by acetone precipitation, and the resulting pellet, as the outer membrane (OM) fraction, was resuspended in 20 mM Tris-HCl (pH 7.4). Protein concentration in each subcellular fraction was evaluated using the BCA protein assay kit. Protein samples from different subcellular fractions were separated by 12% SDS-PAGE gel and then stained with 0.25% Coomassie brilliant blue R250 (Sigma-Aldrich, Burlington, MA, USA) or transferred to 0.45 µm NC membranes and followed by visualization by immunoblotting analysis using monoclonal rabbit anti-AlpA antibody (1:3,000).

### Bacterial growth curve analysis

Bacteria were scraped from agar plates after 48 h of growth and then switched to Brucella broth media with 10% FBS and 1% Iso Vitalex under microaerophilic conditions at 37°C for 16 h. The bacterial suspensions were then diluted to an optical density of 0.05 at 600 nm (OD_600_) and cultivated under microaerophilic conditions at 37°C with shaking at 140 r.p.m. for up to 4 days. The growth of bacteria in the Brucella broth medium was evaluated by measuring the absorbance at OD_600_ with a spectrophotometer during an 84-h cell growth period. Bacterial growth was also assessed using the plate counting method under the above-specified conditions to determine the colony-forming unit (CFU) counts. The experiment was repeated in triplicate to obtain the average values and standard deviation.

### SDS and novobiocin sensitivity assay

Bacteria were scraped from agar plates, cultivated under liquid culture conditions for 36 h, and then diluted to an OD_600_ of 0.6 in Brucella broth media. The bacterial suspensions were added to 6-well plates (2 mL/well) supplemented with the indicated agent (detergent SDS or antibiotic novobiocin) in different concentrations and cultivated under a microaerophilic condition at 37°C with shaking at 140 r.p.m. After growth for 72 h, the absorbance at OD_600_ was measured, and the survival rate was defined as follows: survival rate (%) = (OD_600_ of treatment/OD_600_ of control) ×100. Each experiment was executed in three independent tests to obtain the average results and standard deviation.

### Surface hydrophobicity and autoaggregation assays

Bacteria were scraped from agar plates and grown in Brucella broth media under liquid culture conditions for 48 h. For the surface hydrophobicity assay, bacteria were harvested by centrifugation (4,000* × g*, 10 min, 4°C), resuspended and diluted to OD_600_ of 1 in 5 mL phosphate-urea-magnesium sulfate buffer (PUM: 22.2 g/L K_2_HPO_4_ ×3 H_2_O, 7.26 g/L KH_2_PO_4_, 1.8 g/L urea, 0.02 g/L MgSO_4_ ×7 H_2_O; pH 7.1) supplemented with 400 μL n-Hexadecane. The bacterial suspensions were thoroughly mixed using a vortex for 2 min. The tubes containing the suspensions were then left undisturbed on the bench up for 24 h. After phase separation, the aqueous phase was carefully removed, and the absorbance at OD_600_ was measured at 0 (A_0_) and other tested time points (A_t_). The hydrophobicity index was calculated as follows: the hydrophobicity index = (A_0_–A_t_)/A_0_. For the autoaggregation assay, bacteria were harvested by centrifugation (4,000* × g*, 10 min, 4°C), resuspended, and diluted to an OD_600_ of 1 in 6 mL 1× PBS. The resulting tubes were left standing on the bench up for 24 h. The collection of the aqueous phase samples and the OD_600_ recording were executed as described for the surface hydrophobicity assay. The ratio of autoaggregation was defined as follows: the autoaggregation rate (%) = (A_0_–A_t_)/A_0_ ×100. Each experiment was repeated in triplicate to obtain the average values and standard deviation.

### Cell culture and bacterial infection assay

Gastric AGS cells (ATCC® CRL-1739™, a human gastric adenocarcinoma cell line) were cultivated at 37°C in Ham’s F-12 media (Sigma-Aldrich) supplemented with 10% FBS and 1% penicillin/streptomycin (Gibco). For *H. pylori* infection experiments, the bacteria used were in the stationary growth phase. AGS cells, seeded on 6-well plates (4 × 10^5^ cells/well), were infected at a multiplicity of infection (MOI) of 100 in Ham’s F-12 media containing 10% FBS for 6 h. After co-culture with bacteria, the morphological changes of infected cells were recorded by Olympus I×71research inverted system microscope (Olympus, Tokyo, Japan) with 200× magnification.

### Adhesion assay

Following the bacterial infection process described above, the dish with the bacteria-infected AGS cells was washed three times with PBS to remove non-adherent bacteria. The obtained AGS cells were lysed with 0.1% saponin to release the cell-associated bacteria. The obtained lysates were then serially diluted and plated on sheep blood agar plates. The adhered bacteria were quantified by counting the viable colony forming units (CFU) on agar plates after 72 h of incubation under microaerophilic conditions. Each experiment was repeated in triplicate.

### Internalization assay

Following the bacterial infection process described above, the dish with the bacteria-infected AGS cells was treated with Ham’s F-12 media containing 10% FBS and 100 μg/mL gentamicin, incubated for 1 h to eliminate extracellular bacteria, and washed with PBS. The infected cells were further incubated in Ham’s F-12 media containing 10% FBS with 10 μg/mL gentamicin for 24 h under standard culture conditions. Cell lysate preparation, plating, and incubation were carried out as described in the adhesion assay. The number of internalized bacteria was quantified by counting the CFU on agar plates. Each experiment was repeated in triplicate.

### IL-8 measurement

AGS cells were infected with bacteria (MOI of 100) for 6 h, and the supernatants were collected for IL-8 measurements. IL-8 produced by the infected AGS cells was quantitatively analyzed using a human IL-8 uncoated ELISA kit (Invitrogen) according to the manufacturer’s instructions.

### *Galleria mellonella* virulence assay

*G. mellonella* larvae were applied to study the effect of *HP0858* gene disruption on *H. pylori* virulence. No ethical approval was required to use *G. mellonella* larvae for bacterial infection. *G. mellonella* larvae were maintained in a plastic container in the dark at 10–15 °C while reaching the last larval stage, approximately at a 40-day-old caterpillar stage, with 0.1 and 0.3 g in weight and 2.5 to 3 cm in length. *G. mellonella* larvae were ready for injection with *H. pylori* [31,32]. Bacteria were collected from blood agar plates, placed into 1 ml of PBS, adjusted to an OD_600_ of 1, and then serially diluted in PBS, followed by plating on blood agar plates under microaerophilic conditions to estimate the bacterial concentration accurately. Before the bacterial infection procedure, larvae were acclimated to room temperature without food for 1 day. Ten selected larvae in a group were placed in sterile Petri dishes, decontaminated with 70% ethanol using a sterile cotton swab, followed by injecting 20 µL of bacterial suspensions appropriately diluted in PBS (from 1 × 10^7^ to 1 × 10^4^ CFUs/larva) using a Hamilton syringe into the hemocoel at the last left proleg of the larva. A cohort of ten larvae injected with 20 µL PBS was used as the control group to measure any potential lethal effects of the PBS solution or the injection procedure. After injection, larvae in the Petri dishes were incubated at 37°C in standard aerobic conditions without food, and the survival rate of larvae was monitored and recorded at 24-h intervals for 4 days post-infection. Larvae were considered dead when they changed into dark brown or black color and had no movement or were unresponsive to touch [23,33]. To determine the number of viable bacteria in larvae at 0, 12, 24, 48, and 72 h post-infection, an inoculum of bacterial suspensions, approximately 10^7^ CFUs/larva, suspended in PBS, was injected into *G. mellonella* larvae. The exact inoculum was determined by CFU quantification. After the specified post-infection time, the injection site – specifically the last left proleg – was disinfected with 70% ethanol. A sterile needle was then gently inserted at this site to extract hemolymph. On average, approximately 4 µL of hemolymph was collected from each larva, resulting in a pooled volume of around 40 µL from 10 larvae. The pooled hemolymph was maintained on ice throughout the procedure to ensure sample stability. Subsequently, a 20 µL aliquot was subjected to a 10-fold serial dilution in 1× PBS to lyse hemocytes. The diluted samples were plated onto blood agar and incubated at 37°C for 3 to 4 days, after which the bacterial load per larva was quantified by counting CFUs.

### Statistics analysis

All statistical analyses were performed using GraphPad Prism version 8.3.0.538 (GraphPad Software, San Diego, CA, USA). Each experiment was conducted with at least three independent repetitions. Survival curves were plotted using the Kaplan-Meier method, and the differences in survival were calculated using the log-rank (Mantel-Cox) test for multiple comparisons. Error bars represent the standard deviations (SD), and the probability values (*p* values, p) are calculated using an unpaired, two-tailed Student’s *t*-test. Asterisks indicate the data with statistical significance (* p < 0.05, ** p < 0.01, *** p < 0.001, **** p < 0.0001).

## Results

### Sequence analysis of HP0858 protein in *H.* *pylori* strain 26,695

**Figure 2.**
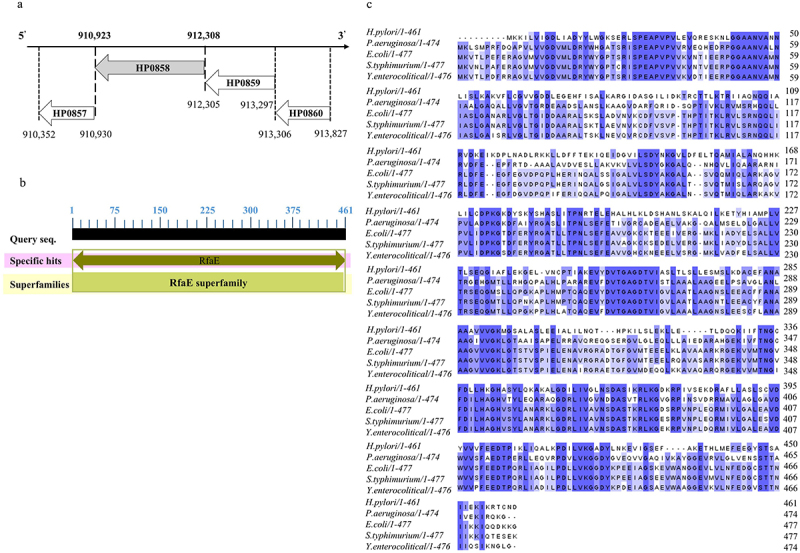
The bioinformatic analysis of *HP0858* gene and its encoding protein in *H. pylori* 26,695 strain. (a) The gene locus of *HP0858*. (b) The prediction of conserved domains in the HP0858 protein. The data were obtained from the NCBI (https://www.ncbi.nlm.nih.gov/pmc/) accessed on 12 November 2022. (c) Multiple sequence alignment of HP0858 protein and its homologues with D-*glycero*-α, β-D-*manno*-heptose 7-phosphate kinase/D-*glycero*-β-D-*manno*-heptose 1-phosphate adenosyltransferase activity (RfaE/hlde): *Helicobacter pylori* 26,695 [O25529], *Pseudomonas aeruginosa* PAO1 [Q9HUG9], *Escherichia coli* K-12 [P76658], *Salmonella typhimurium* LT2 [Q7CPR9] and *Yersinia enterocolitica* 8081 [A1JQV6]. The numbers on the right side of the figure indicate the amino acid positions. The gaps are represented by dashes. The identical and similar residues were marked with dark and light blue colors, respectively, indicating different degrees of similarity. Multiple sequence alignment was performed using the ClustalW2 program (https://www.ebi.acuk/Tools/msa/clustalw2/) accessed on 12 November 2022. Shading was obtained using the jalview server (https://www.jalview.org/) accessed on 12 November 2022.

The *HP0858* coding sequence is located at the minus strand of the *H. pylori* genome from 910,923 to 912,308, flanked by the *HP0857* gene and the *HP0859* gene in strain 26,695 (Figure 2(a)). The entire length of the *HP0858* gene is 1,386 bps and can be translated into a protein with 461 amino acid residues. The conserved domain of the HP0858 protein is found in the members of the RfaE superfamily (Figure 2(b)). The amino acid sequences of HP0858 homologous proteins from different species of Gram-negative bacteria were aligned using Clustal Omega, and the obtained results were colored using Jalview. According to the results, HP0858 protein shows high sequence homology with RfaE, also called HldE, from other Gram-negative bacteria (Table 3 and Figure 2(c)). This result indicates that HP0858 protein could be an RfaE that contains both kinase and adenylyltransferase activity and plays a crucial role in the biosynthesis of heptose and, thus, is necessary for the structural integrity of the LPS core region in *H. pylori* (Figure 1).

**Table 3.** Sequence comparison of *H. pylori* HP0858 protein from *H. pylori* wild-type strain 26,695 with the corresponding homologues from other gram-negative bacteria.

	Species	UniProt AC^1^	Identities (%)	Similarity (%)	Length
HP0858 (RfaE/HldE)	*Helicobacter pylori* 26,695	O25529	100%	100%	461 aa
	*Pseudomonas aeruginosa* PAO1	Q9HUG9	42%	60%	474 aa
	*Escherichia coli* K-12	P76658	41%	59%	477 aa
	*Salmonella typhimurium* LT2	Q7CPR9	41%	58%	477 aa
	*Yersinia enterocolitica* 8081	A1JQV6	40%	59%	476 aa

^1^An accession number (AC) that is assigned to each sequence upon inclusion into UniProt (https://www.uniprot.org/) accessed on 12 November 2022; aa, amino acid.

### Gene disruption in *HP0858* results in a truncated LPS structure

**Figure 3.**
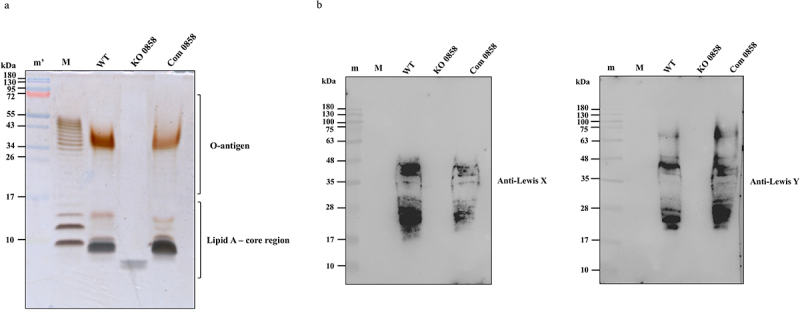
Effects of the *HP0858* gene-disrupted mutation on LPS structure and Lewis antigen appearance. The LPS profiles from 26,695 strain WT, the *HP0858* knockout mutant, and its corresponding complemented strain were examined by (a) 15% tricine-PAGE with silver staining or (b) immunoblotting analysis with anti-Lewis X and anti-Lewis Y antibodies. The relative positions of the O-antigen and lipid A-core region were marked with braces. WT, wild-type strain; KO, knockout mutant; Com, complemented strain.; M, LPS from *E. coli* O111:B4; m’, the protein ladder used was the TriColor prestained protein ladder (10–180 kDa; catalog number AGEL3523), supplied by Assay Genie (Dublin, Ireland); m, the protein marker used was the AccuRuler RGB prestained protein ladder (10–180 kDa; catalog number 02101–250), supplied by MaestroGen Inc. (Hsinchu city, Taiwan).

*H. pylori* strain 26,695 [KE26695] (ATCC 700,392; CagA^+^, VacA^+^) was used in this study for all analyses. This isolate is non-motile and non-flagellated [34]. Supplementary Figure S1 demonstrates that the *H. pylori* strain 26,695 and its derived mutants tested in this study consistently remained near the inoculation zone over the 6-day culture period, providing evidence of their lack of motility. To investigate the role of *HP0858* in LPS biosynthesis, the *HP0858* knockout mutant and its corresponding complemented control strain were constructed. The *HP0858* gene in genomic *H. pylori* DNA was disrupted by inserting a chloramphenicol resistance cassette (Cm^r^) to generate the *HP0858* knockout mutant. The *HP0858* complemented strain was constructed by inserting a DNA fragment carrying the promoter region of the *HP1563* gene in front of the full-length *HP0858* gene into the *rdxA* locus of the *HP0858* knockout mutant through natural transformation. The successful generation of these mutants was first confirmed by PCR amplification of the target genes and then further verified through gene sequencing (data not shown). To examine the effects of *HP0858* gene disruption on LPS expression, LPS from different *H. pylori* strains were isolated and analyzed using silver staining and immunoblotting with anti-Lewis X and Y antibodies. As shown in Figure 3(a), the *HP0858* knockout mutant had a truncated LPS structure, which contained only a part of the Lipid A-core oligosaccharide and lost all of the O-antigen domain while separating on a tricine gel with silver staining. In addition, while testing with immunoblotting analysis to examine the presence of Lewis antigens, the signals of Lewis X and Lewis Y antigens were utterly missing (Figure 3(b)). In contrast, the above LPS defects were fully restored in the *HP0858* complemented strain (Figures 3(a,b)). Our results suggest that the protein encoded by the *HP0858* gene plays a crucial role in the formation of LPS core oligosaccharide, and the disruption of *HP0858* involved in the heptose biosynthetic pathway not only affects the length of *H. pylori* LPS but also causes a complete loss of Lewis antigen moieties.

### *HP0858* gene-disrupted mutation slightly affects bacterial growth and causes hypersensitivity to detergent SDS and antibiotic novobiocin

**Figure 4.**
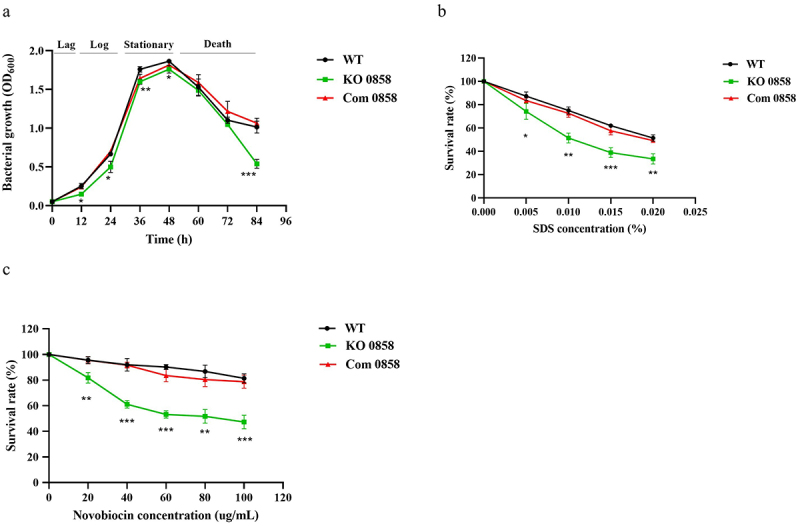
Effects of the *HP0858* gene-disrupted mutation on *H. pylori* growth, sensitivity to the detergent SDS, and resistance to the antibiotic novobiocin. (a) The growth curves of 26,695 strain WT, the *HP0858* knockout mutant, and its corresponding complemented strain. The growth data were measured with a spectrophotometer by recording the OD_600_ values at different time points. The data are displayed as the average OD_600_ values obtained from triplicate tests with statistical analysis (unpaired, two-tailed Student’s t-test; *p < 0.05, **p < 0.01, ***p < 0.001). (b) SDS sensitivity and (c) novobiocin resistance of the tested *H. pylori* strains. The effects of the *HP0858* mutation on SDS sensitivity and novobiocin resistance of *H. pylori* were evaluated by the survival rate, which was calculated from the ratio of the OD_600_ of bacteria treated with/without various concentrations of SDS and novobiocin. The data displayed were obtained from triplicate tests with statistical analysis (unpaired, two-tailed Student’s t-test; *p < 0.05, **p < 0.01, ***p < 0.001, ****p < 0.0001). WT, wild-type strain; KO, knockout mutant; Com, complemented strain.

To investigate if a deficiency in the heptose biosynthetic pathway of LPS could have an impact on the bacterial growth rate of *H. pylori*, the bacterial growth of the *H. pylori* 26,695 WT strain WT, the *HP0858* knockout mutant, and the *HP0858* complemented strain was measured at OD_600_ by a spectrophotometer at different time points up to 84 h. The growth curves of all tested bacteria entered the log phase after 12 h of cultivation, reached the stationary phase after 36 h of growth, and then went into the death phase after 48 h of growth, even though a slight reduction in the growth rate was observed for the *HP0858* knockout mutant (Figure 4(a)). These results indicate that the disruption of the *HP0858* gene involved in the heptose biosynthetic pathway of LPS formation does not cause a dramatic effect on the growth of *H. pylori* at the time points tested.

LPS in Gram-negative bacteria is thought to maintain the structural integrity of bacterial outer membranes and provide a barrier against the entry of toxic hydrophobic compounds. Previous research from our laboratory and other laboratories has already found that Gram-negative mutants defective in heptose biosynthesis for the LPS construction display increasing sensitivity to low concentrations of antibiotics and detergents [25–27,35]. To investigate whether the disruption of the *HP0858* gene would affect the resistance of *H. pylori* to detergents and hydrophobic antibiotics, SDS and novobiocin, respectively, were used as agents to induce bacterial growth stress. As shown in Figures 4(b,c), the *HP0858* gene-disrupted mutant exhibited hypersensitivity to SDS and novobiocin, and the *HP0858* complemented strain restored most of the WT resistance level to these agents. These results indicate that the HP0858 protein involved in the heptose biosynthetic pathway is essential in constructing the *H. pylori* LPS core structure, which is critical in bacterial resistance to the entry of toxic hydrophobic molecules.

### The *HP0858* gene-disrupted mutation significantly changes the surface properties of *H.* *pylori*

**Figure 5.**
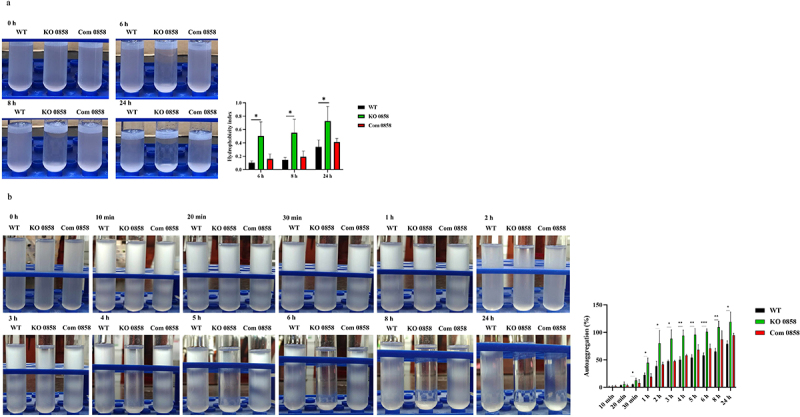
Effects of the *HP0858* gene-disrupted mutation on the surface properties of *H. pylori*. The bacterial suspensions were incubated with (a) n-hexadecane for the surface hydrophobicity analysis and (b) phosphate-buffered saline (PBS) for the autoaggregation assay. The hydrophobicity index and autoaggregation percentage were calculated according to the procedures described in the materials and methods section. They represent the cell surface hydrophobicity and bacterial autoaggregation, respectively. The OD_600_ value obtained from the zero-time point was employed as a control for background subtraction. Data displayed are derived from triplicate tests with statistical analysis (unpaired, two-tailed Student’s t-test; *p < 0.05, **p < 0.01, ***p < 0.001). WT, wild-type strain; KO, knockout mutant; Com, complemented strain.

The disappearance of core oligosaccharides in LPS has been demonstrated to increase the cell surface hydrophobicity of *E. coli* [36]. The surface hydrophobicity and autoaggregation assays were applied to test the effects of *HP0858* gene disruption on the surface properties of *H. pylori*. The surface hydrophobicity assay applied here measured the hydrophobic surface properties of bacteria by testing their ability to adhere to a hydrocarbon, n-Hexadecane. As shown in Figure 5(a), a “creamy and white” layer was formed at the top of the tubes after phase separation. This layer is an oil-in-water emulsion consisting of n-Hexadecane droplets and can be laden with adherent bacteria if bacteria’s adhesion to n-Hexadecane occurs. A reduction in the OD_600_ measurement of the lower aqueous phase was used to evaluate the cell surface hydrophobicity. The result showed that the *HP0858* knockout mutant significantly increased its surface hydrophobicity compared to the WT strain at all the tested time points (6 h, 8 h, and 24 h). In addition, this mutant’s autoaggregation tendency also displayed a similar trend to that found in the surface hydrophobicity assay (Figure 5(b)). The *HP0858* knockout mutant strain started to autoaggregate after 30 min of incubation. As for the *HP0858* complemented strain, it displayed a WT-like level of surface hydrophobicity and autoaggregation.

### The *HP0858* gene-disrupted mutation has significant impacts on H. pylori virulence

**Figure 6.**
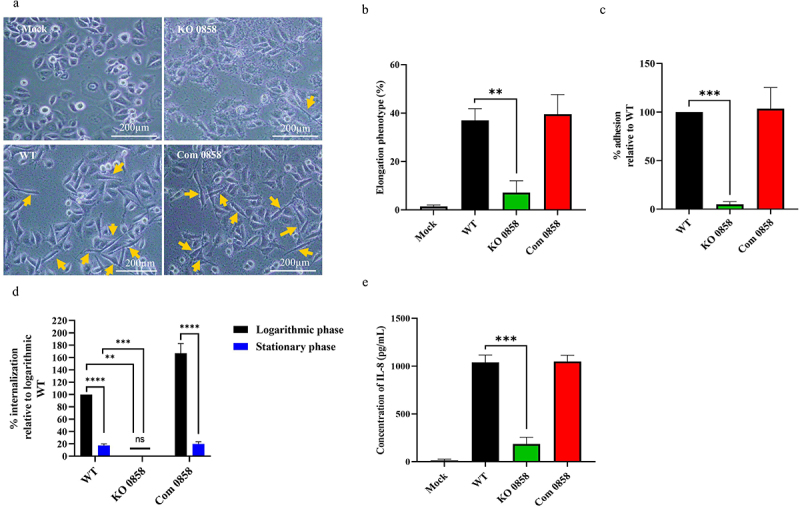
Effects of the *HP0858* gene-disrupted mutation on bacterial virulence. (a) Morphological changes of the AGS cells infected by different *H. pylori* strains. The morphological changes of the AGS cells infected by various *H. pylori* strains were observed by phase-contrast microscopy with 200× magnification. The AGS cells displaying an elongated character (hummingbird phenotype) were indicated by arrows (scale bar = 200 µm). (b) Quantification of the AGS cells with the elongated phenotype after *H. pylori* infection. The percentage of elongated cells was quantified from 10 different 0.25 mm^2^ fields. The data shown were obtained from triplicate tests with statistical analysis (unpaired, two-tailed Student’s t-test; **p < 0.01). (c) Adhesion of various *H. pylori* strains to AGS cells during bacterial infection. Various *H. pylori* strains were co-incubated with AGS cells at an MOI (multiplicity of infection) of 100. The adhesion of different mutants assayed by the viable CFU counting method was compared to that of 26,695 strain WT to acquire the relative percentage of bacterial adhesion. The data shown were obtained from triplicate tests with statistical analysis (unpaired, two-tailed Student’s t-test; ***p < 0.001). (d) Internalization of various *H. pylori* strains into AGS cells during bacterial infection. Various *H. pylori* strains in the logarithmic phase (cultured for 27 hours) and stationary phase (cultured for 48 hours) were co-incubated with AGS cells at an MOI of 100. Using the viable CFU counting method, the internalization efficiency of each strain was assessed and compared to that of the 26,695 strain WT in the logarithmic phase to determine the relative percentage of bacterial internalization. The data presented were derived from triplicate trials with statistical analysis (unpaired, two-tailed Student’s t-test; ns (non-significant) *p* > 0.05, **p < 0.01, ***p < 0.001, ****p < 0.0001). (e) The amount of IL-8 production from AGS cells after being infected by various *H. pylori* strains. The data presented were derived from triplicate trials with statistical analysis (unpaired, two-tailed Student’s t-test; ***p < 0.001). WT, wild-type strain; KO, knockout mutant; Com, complemented strain.

*H.* *pylori* has been reported to infect AGS cells and cause a morphological change called the “hummingbird or elongation phenotype” [37–40]. To study the role of HP0858 upon infection, AGS cells were co-incubated with the various produced strains for 6 h. Figures 6(a,b) show that approximately 37% and 39% of AGS cells displayed an elongated phenotype after being infected with WT and the *HP0858* complemented strain, respectively. Conversely, less than 10% of elongated AGS cells were present in those infected by the *HP0858* knockout mutant. These results suggest that the *HP0858* gene-disrupted mutation could reduce the infection abilities of *H. pylori*.

*H. pylori’*s adhesion to gastric epithelial cells is crucial in successfully colonizing the gastric mucosa. The difference in the adhesion capabilities of *H. pylori* WT and the LPS core-disrupted *HP0858* knockout mutant toward AGS cells was investigated. The findings revealed that approximately 50.67% of the WT *H. pylori* inoculum adhered to AGS cells following a 6-h infection period using stationary-phase bacteria cultured for 48 h (data not shown). Under the same experimental conditions, the *HP0858* knockout mutant exhibited a significantly reduced adhesion rate compared to the WT strain (Figure 6(c)). In contrast, the adhesion level of its corresponding complemented strain to AGS cells was restored to the WT level. This finding indicates that the heptose biosynthesis-related HP0858 plays a role in the adhesion capabilities of this pathogenic bacterium. On the other hand, *H. pylori* is considered an extracellular pathogen and does not invade host cells during bacterial infection. However, some evidence suggests that a limited number of these bacteria can still enter host cells under certain circumstances [41]. Therefore, the effect of the LPS core-related *HP0858* mutation on the ability of *H. pylori* to invade gastric cells was also tested here. When stationary-phase bacteria (cultured for 48 h) were tested, approximately 0.009% of the WT *H. pylori* inoculum was internalized into AGS cells after 6 h of infection (data not shown). Under the same conditions, the *HP0858* knockout mutant exhibited a significantly lower percentage of internalization (Figure 6(d)). However, the invasion rate of the *HP0858* complemented strain was restored to the WT level. Interestingly, when logarithmic-phase bacteria (cultured for 27 h) were used in the internalization assay, the *HP0858* knockout mutant again exhibited a pronounced decrease in internalization efficiency compared to the WT strain, mirroring the trend observed with stationary-phase bacteria. Two notable observations emerged from this experiment: (1) the WT strain in the logarithmic phase demonstrated approximately a 6-fold increase in internalization (about 0.053% of the initial inoculum) relative to the stationary phase (approximately 0.009%); and (2) the complemented strain surpassed the WT by 64% in internalization efficiency (0.087% vs. 0.053% of the original inoculum) under logarithmic-phase conditions. While both findings reached statistical significance, their biological relevance may be limited given the overall low internalization levels observed under these experimental conditions.

Given the significant impact of *HP0858* disruption on *H. pylori* adhesion, it was pertinent to investigate its effect on interleukin-8 (IL-8) secretion – a response primarily dependent on direct contact between *H. pylori* and gastric epithelial cells. Moreover, prior studies have identified ADP-heptose as a novel pathogen-associated molecular pattern (PAMP) in Gram-negative bacteria, capable of stimulating IL-8 production in human epithelial cells [19,42]. Accordingly, we compared IL-8 secretion levels in AGS cells infected with either the *H. pylori* WT strain or the *HP0858* knockout mutant. As illustrated in Figure 6(e), the *HP0858* mutant elicited significantly lower IL-8 secretion than the WT strain. This finding strongly suggests that the reduced IL-8 response is primarily attributable to the adhesion deficiency caused by *HP0858* disruption. Additionally, it is plausible that intermediates in the HP0858-associated heptose biosynthesis pathway also contribute to IL-8 induction in gastric epithelial cells. Collectively, these results indicate that impairment of the HP0858-involved heptose biosynthetic pathway markedly compromises key virulence traits of *H. pylori*.

### *HP0858* gene-disrupted mutation attenuates *H.* *pylori* virulence in the *G.* *mellonella* infection model

**Figure 7.**
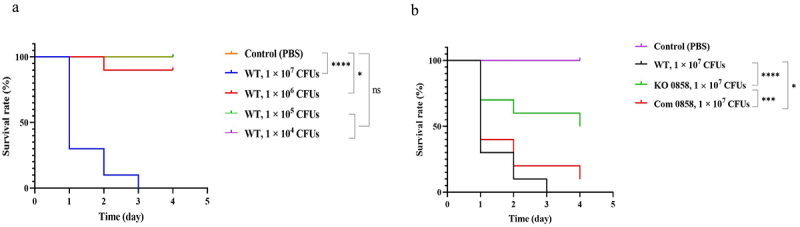
Survival curves of *G. mellonella* larvae after infection with various *H. pylori* strains. (a) Kaplan-meier survival curves of *G. mellonella* larvae following infection with increasing concentrations of *H. pylori* 26,695 strain WT. *G. mellonella* larvae were injected with 1 × 10^4^, 1 × 10^5^, 1 × 10^6^, and 1 × 10^7^ CFUs/larva and incubated at 37°C for up to 4 days to obtain Kaplan-meier survival curves. (b) Kaplan-meier survival curves of *G. mellonella* larvae following infection with various *H. pylori* strains. *G. mellonella* larvae were injected with 1 × 10^7^ CFUs/larva of the 26,695 strain WT, the *HP0858* gene-disrupted mutant and the *HP0858* complemented strain and incubated at 37°C up to 4 days to obtain Kaplan-Meier survival curves. The data shown represent the percentage of survival rate from three independent experiments. Differences in survival curves were calculated using the log-rank (mantel-cox) test for multiple comparisons. Differences were considered statistically significant at ****p < 0.0001, **p < 0.01, *p < 0.05, ns: no significant difference. PBS, phosphate-buffered saline; WT, wild-type strain; KO, knockout mutant; Com, complemented strain.

The *G. mellonella* (greater wax moth larvae) infection model has emerged as a valuable tool in microbiology research for assessing microbial virulence, including *H. pylori*. These larvae possess an immune system that shares key features with the human innate immune system, such as cellular and humoral responses [43]. In this study, *G. mellonella* larvae were utilized as an *in vivo* infection model to explore a distinct aspect of how HP0858 disruption influences the host interactions compared to previously studied epithelial cell models. Initially, a range of bacterial dosages (10^4^, 10^5^, 10^6^, and 10^7^ CFUs/larva) of the 26,695 WT strain was injected into *G. mellonella* larvae. The infected larvae were then incubated at 37 °C for up to 4 days to determine the optimal dosage that could cause more than 50% larval mortality [23]. The larvae were considered dead if they did not show a noticeable response to external stimuli. As displayed in Figure 7(a), the *H. pylori* 26,695 WT strain caused larval death in a time- and dose-dependent manner. No mortality was found in the control group’s larvae, which were only injected with PBS buffer alone. At the dosage of 1 × 10^7^ CFUs/larva, approximately 30% of the larvae still survived after the first day of post-infection, and no larvae were alive after the third day of post-infection, which clearly showed the difference in virulence potential compared to that of other concentrations used in the test (*p* < 0.0001). Therefore, the bacterial dosage of 1 × 10^7^ CFUs/larva was chosen as the optimal dosage of injected bacteria for the following experiments to evaluate the effect of the *HP0858*-disrupted mutation on the virulence of *H. pylori* infection. As shown in Figure 7(b), larvae infected with the *HP0858* gene-disrupted mutant showed a 70% survival rate after the first day of post-infection. The survival rate was slightly reduced to 60% and 50% after the second and the fourth days of post-infection, respectively, significantly lower than those observed in larvae infected with the 26,695 WT strain and the *HP0858* complemented strain. As control, no mortality was observed in *G. mellonella* larvae injected with PBS during the experiment.

Next, the bacterial load in the hemolymph of *Galleria mellonella* larvae was evaluated at 0, 12, 24, 48, and 72 h following a 30-min post-injection period with the various *H. pylori* strains, using the viable CFU quantification. As detailed in Table 4, viable bacteria were successfully recovered from the hemolymph of larvae infected with all tested *H. pylori* strains at the initial time (30 min post-injection). Across all strains, a gradual decline in viable bacterial load was observed over time compared to the original inoculum. Notably, viable bacterial loads for larvae injected with the 26,695 strain WT and the *HP0858* complemented strain remained detectable at 72 h post-injection. In stark contrast, no viable bacteria were recovered from larvae injected with the *HP0858* gene-disrupted mutant as early as 48 h post-infection. Furthermore, no bacterial colonies were detected in larvae injected with PBS. These results suggest that the *HP0858* gene-disrupted mutation caused attenuation of bacterial virulence in the *G. mellonella* infection model.

**Table 4.** Viable counts (CFUs; means ±SD) of various *H. pylori* strains in the hemolymph of *G. mellonella* at 0, 12, 24, 48, and 72 h post-infection.

Strains	Ti^2^	T0^1^	T12	T24	T48	T72
PBS (1Χ)	0	0	0	0	0	0
WT	1.6 (±0.6) × $10^{7}$	1.4 (±0.6) × $10^{7}$	1.8 (±0.7) × $10^{6}$	2.9 (±1.4) × $10^{4}$	1.4 (±0.6) × $10^{4}$	1.7 (±0.6) × $10^{1}$
KO 0858	1.5 (±0.3) × $10^{7}$	1.1 (±0.1) × $10^{7}$	2.3 (±0.6) × $10^{3}$	1.7 (±0.6) × $10^{1}$	0	0
Com 0858	1.5 (±0.6) × $10^{7}$	1.3 (±0.6) × $10^{7}$	1.7 (±0.4) × $10^{4}$	1.9 (±0.7) × $10^{3}$	1.7 (±0.6) × $10^{3}$	0.7 (±0.6) × $10^{1}$

Ti is the original time for larvae infection ^1^Time 0 corresponds to 30 min after larvae infection.

### The *HP0858* gene-encoded enzyme is involved in the glycosylation process of adhesin AlpA in the *H.* *pylori* 26,695 strain

**Figure 8.**
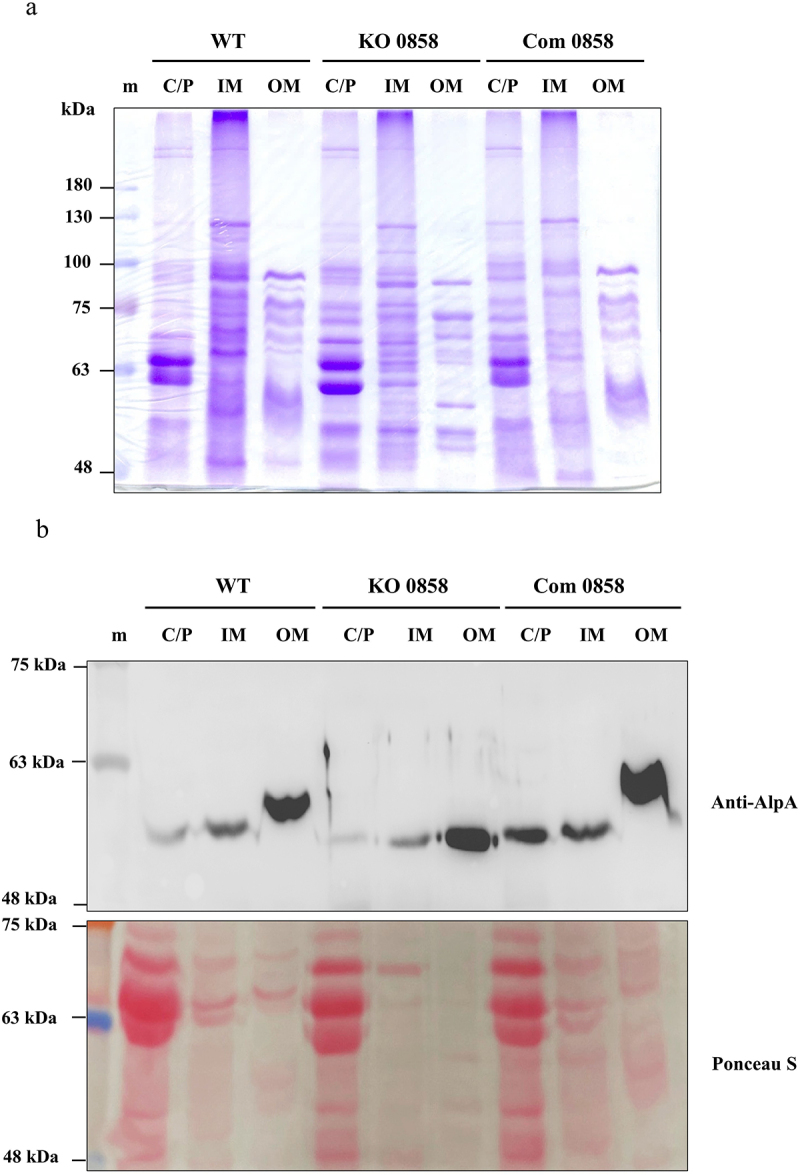
Effects of the *HP0858* gene-disrupted mutation on the glycosylation of adhesin AlpA. (a) The protein profiles of the subcellular fraction samples (10 μg/lane) from the tested bacterial strains were analyzed by staining with 0.25% coomassie brilliant blue R250. (b) The electrophoretic mobility of adhesin AlpA in different subcellular fractions from the tested bacterial strains was analyzed by immunoblotting analysis with anti-AlpA antibody. Protein samples were collected from the cytoplasm/periplasm (C/P, 80 μg), the inner membrane (IM, 60 μg), and the outer membrane (OM, 10 μg). The result of ponceau S staining was used as the loading control for comparison. WT, wild-type strain; KO, knockout mutant; Com, complemented strain; m, the protein marker used was the AccuRuler RGB prestained protein ladder (10–180 kDa; catalog number 02101–250), supplied by MaestroGen Inc. (Hsinchu city, Taiwan).

We recently demonstrated that several major adhesins, including AlpA in the *H. pylori* 26,695 strain, are modified with glycans, and the glycosylation process of these adhesins is associated with a general protein glycosylation system, which partially shares the enzymes from the LPS biosynthetic pathway [44]. We therefore tested if the gene-disrupted mutation in *HP0858* would affect the glycosylation of AlpA, a well-known adhesin of *H. pylori*. As illustrated in Figure 8(a), numerous proteins are present in the outer membrane (OM) fractions, displaying unique profiles that differ noticeably from those observed in the cytoplasm/periplasm (C/P) and the inner membrane (IM) fractions. This distinction indicates that the subcellular fractionation process was carried out effectively. Interestingly, the electrophoretic mobility of AlpA indeed displayed an observable two-step molecular weight upshift pattern first from the cytoplasm to the inner membrane and then from the inner membrane to the outer membrane, a typical phenomenon for glycosylated adhesins in *H. pylori*, which was observed both in WT and the *HP0858* complemented strains (Figure 8(b)). In contrast, this molecular weight upshift phenomenon disappeared in the *HP0858* knockout mutant. This observation supports our recent finding and suggests that the *HP0858* gene-encoded enzyme is also a component of the general protein glycosylation system just discovered in *H. pylori*.

## Discussion

Although most of the *H. pylori* enzymes associated with the biosynthesis of lipid A and O-antigen have been identified and characterized, some of the enzymes involved in the biosynthesis of the core region of LPS remain to be explored [20]. Our group previously conducted studies on the *HP0857*, *HP0859*, and *HP0860* genes and demonstrated that they all play essential roles in the heptose biosynthetic pathway for constructing the core region of *H. pylori* LPS [25–27]. In this study, the remaining *HP0858* gene in the gene cluster for heptose biosynthesis was characterized by several molecular techniques.

The sequence analysis of the HP0858 protein compared to several Gram-negative bacterial homologs indicates that HP0858 is highly similar to the members of the RfaE superfamily that are known to possess both kinase and adenylyltransferase activities required for heptose biosynthesis. Here we could show that the *HP0858* mutant not only exhibits a severe truncated LPS structure in the core region but also displays a complete loss of Lewis antigens, proving that the encoded enzyme is indeed involved in heptose biosynthesis and thus is critical for the formation of *H. pylori* LPS. In addition, this mutant increases the sensitivity to detergent SDS and antibiotic novobiocin, supporting the well-recognized concept that the LPS core plays an essential role in providing a protective barrier to restrict the entrance of toxic hydrophobic compounds like bile salts, detergents, lipophilic antibiotics, and other poisonous substances that might harm the bacterium [45,46].

Specific LPS metabolites derived from heptose biosynthesis are acknowledged as novel pathogen-associated molecular patterns (PAMPs) identified in Gram-negative bacteria [47]. Instead of the initially recognized HBP (the product of the second enzymatic step in the ADP-heptose biosynthetic pathway) [16–18], ADP-heptose, the fourth-step product of the pathway as mentioned above, is currently recognized as the predominant PAMP of *H. pylori* in the activation of the NF-κB pathway in human epithelial and phagocytic cells through the ALPK1-TIFA axis and thus induce the secretion of the proinflammatory cytokine IL-8 [19,48]. In addition to triggering proinflammatory signal transduction in host cells, the induction of NF-κB signaling by ADP-heptose sensing during *H. pylori* infection has also been reported to trigger RNA-loop-mediated DNA replication stress and promote DNA double-strand breaks (DSBs) [49]. The above genotoxic stress, joined with erroneous DNA repair in infected cells, can increase genome instability and thus promote gastric cancer development. The current study, consistent with earlier findings [19], reveals that disrupting *HP0858* expression, which impairs ADP-heptose production, significantly decreases IL-8 secretion in AGS cells during infection. However, our team also observed that AGS cells infected with an *H. pylori* mutant lacking *HP0860* expression still produced notable amounts of IL-8 [27]. We explained this unexpected outcome by proposing that other phosphatase activities within *H. pylori* may partially compensate for the loss of *HP0860* expression in the mutant strain.

Hauke et al. recently demonstrated that, compared to other genes involved in the biosynthesis of heptose metabolites, the *RfaE/HldE/HP0858* gene and its corresponding enzyme exhibit notable sequence variability and expression differences among *H. pylori* strains [50]. They further revealed that the heptose gene cluster’s expression varies within and between strains, influenced by factors such as host cell contact, the presence of the *cag* pathogenicity island (*cag*PAI), and the carbon starvation regulator A (CsrA). Interestingly, using an approach that reconstituted the heptose biosynthetic pathway and analyzed the resulting metabolites both *in vitro* and in *H. pylori* lysates, the researchers identified a third heptose metabolite – likely heptose-1-monophosphate (HMP-1) – in addition to ADP-heptose and HBP. This metabolite was characterized as a novel proinflammatory compound produced by *H. pylori* [50]. RfaE/HldE/HP0858 is a bifunctional enzyme comprising two domains, d1 and d2, responsible for kinase activity and adenylyltransferase activity, respectively, which are essential for the second and fourth steps in the heptose biosynthetic pathway. Unexpectedly, the same group found that the d1 domain also exhibits phosphatase activity *in vitro*, enabling it to produce HMP-1 during the first two steps of heptose biosynthesis without requiring the third-step enzyme GmhB/HP0860. This discovery provides a possible explanation for the observation that AGS cells infected with the HP0860 knockout mutant can still secrete significant levels of IL-8.

Our study observed a notable increase in surface hydrophobicity in the *HP0858* knockout mutant, accompanied by a tendency for the bacteria to autoaggregate. Autoaggregation can introduce variability in OD_600_ readings, potentially distorting their reflection of bacterial growth. To address this, we reevaluated the growth curve using the CFU counting method along with OD_600_ measurements. The results were then compared using semi-logarithmic graphs. As shown in Supplementary Figure S2, the absence of data collection during the first 12 h of culture prevented the identification of a clear lag phase in both growth curves. Interestingly, the two methods yielded similar growth trends during the stationary phase (36–48 h) and death phase (48–84 h) but diverged significantly in the logarithmic phase. The OD_600_-based growth curve displayed a steep, linear increase, whereas the CFU-derived curve was more level. Moreover, during this stage, the OD_600_ measurements revealed more pronounced differences between the WT strain and the *HP0858* knockout mutant than the CFU method. It is worth noting that CFU counts may underestimate viable cell numbers when cells experience stress, while OD_600_ readings might overestimate growth by including non-viable cells. However, the observed discrepancies suggest that the autoaggregation in the *HP0858* knockout mutant could lead to reduced OD_600_ readings, resulting in an underestimation of its growth. Although statistical analysis shows that the mutant grows slightly slower than the WT strain at specific time points, these differences are unlikely to carry significant biological implications.

Beyond the direct effects outlined in the main text, the autoaggregation phenotype resulting from *HP0858* disruption could also indirectly influence specific findings examined in this study. Aggregating populations of the *HP0858* knockout mutant are expected to be less efficient at dispersing, which could diminish their ability to colonize and adhere to host epithelial cells effectively. This reduced dispersion may lead to localized bacterial clustering, limiting the bacteria’s capacity to spread across the gastric mucosa. Consequently, this can decrease the efficiency of bacterial adherence and invasion, which is crucial for establishing infection and initiating host-pathogen interactions. Without efficient adherence, key virulence factors such as adhesins (e.g. BabA and SabA) may not optimally engage with host receptors, thereby reducing the infection’s overall pathogenic impact. Furthermore, a reduced direct interaction due to bacterial aggregation may impair CagA translocation through the T4SS, dampening the morphological changes typically induced by this effector protein. The T4SS activity is also crucial for the induction of pro-inflammatory cytokine IL-8 via NF-κB activation. Aggregation, by reducing T4SS functionality, may lead to lower levels of IL-8 production, potentially moderating the inflammatory response.

Consistent with the findings for the *HP0858* knockout mutant in this study, our previous research demonstrated that disrupting other heptose biosynthesis-related genes (*HP0857* (coding for an isomerase), *HP0859* (coding for an epimerase), and *HP0860* (coding for a phosphatase)) not only truncates the LPS structure, thereby eliminating the presence of Lewis X and/or Lewis Y antigens, but also reduces specific phenotypes such as adhesion, antibiotic and detergent resistance, and virulence [25–27]. This alignment suggests that the phenotypic changes observed in this study are not exclusive to HP0858 but are shared among other enzymes involved in heptose biosynthesis. Interestingly, we recently identified similar phenotypic defects, including increased surface hydrophobicity and autoaggregation, in mutants with disruptions in *HP0044* and *HP1275* [51]. These genes encode enzymes critical for fucose biosynthesis. Fucose is a key component of the Lewis X and Lewis Y antigens in the LPS O-antigen of *H. pylori*. Furthermore, fucose is part of a conserved trisaccharide (-GlcNAc-Fuc-DD-Hep-) known as “Trio,” which connects the core oligosaccharide to the most recently redefined O-antigen structure. Based on these findings, it is plausible to hypothesize that the absence of Lewis X/Lewis Y antigens, or more broadly, the disruption of the O-antigen structure, underlies the phenotypic defects observed in these *H. pylori* mutants.

Glycosylation is a type of post-translational modification that plays diverse roles in protein function and contributes to the complexity of a cell’s proteome. In Gram-negative bacteria, an increasing number of glycoproteins have been identified, many of which act as virulence factors. For instance, AIDA-I, an adhesin involved in the diffuse adherence of diarrhea-causing *E. coli* strain 2787 (O126:H27), has been recognized as a glycoprotein [52]. Benz and Schmidt revealed that AIDA-I is modified with heptose residues, and its glycosylated form significantly enhances bacterial adherence to human cell lines. In contrast, the non-glycosylated version lacks this ability. Recently, we demonstrated that *H. pylori* possesses a general protein glycosylation system responsible for modifying several key adhesins. This modification system is crucial for ensuring adhesins’ stability and protease resistance and enhancing their binding capabilities [44]. The enzymes linked to this newly discovered system partially overlap with those involved in the biosynthesis of LPS. In this research, we identified a connection between LPS biosynthesis and the potential glycosylation of the adhesin AlpA, mediated by a sugar motif containing heptose residues or their derivatives. Based on this relationship, it is proposed that AlpA undergoes glycosylation through a general protein glycosylation pathway associated with LPS biosynthesis, with HP0858 being one of the enzymes involved. The *HP0858*–disrupted mutant reduces bacterial adhesion and internalization abilities when infecting AGS cells, and the infected AGS cells show a lower degree of the “elongation phenotype” compared to those infected with the *H. pylori* 26,695 WT strain. These altered characteristics in the HP0858-deficient mutant can be attributed to two potential mechanisms: (1) Direct impact: LPS functions as an adhesion molecule in *H. pylori*, and disruption of heptose biosynthesis compromises the structural integrity of LPS, significantly impairing the bacterium’s adhesion ability and virulence; (2) Indirect impact: Loss of HP0858 expression may prevent the glycosylation of key adhesins like AlpA, hindering *H. pylori* from effectively binding to host cells and delivering virulence factors such as CagA. These two mechanisms are not mutually exclusive and may collectively contribute to the observed phenotypic changes.

Notably, the molecular size of AlpA exhibited a distinct two-step upshift as it transitioned from the cytoplasm to the inner membrane and eventually to the outer membrane of *H. pylori*. This progressive size increase suggests that the glycosylation of AlpA likely begins during or shortly after its transport across the inner membrane into the periplasm and is completed before its integration into the outer membrane. Based on this observation, it is reasonable to infer that AlpA in the outer membrane is fully glycosylated. In contrast, the form detected in the inner membrane represents a partially glycosylated intermediate state. However, an alternative explanation cannot be ruled out entirely: a secondary glycosylation system located within the periplasm might finalize the glycosylation process for AlpA. Further research is required to confirm this possibility.

In this study, we utilized *Galleria mellonella* (greater wax moth) larvae as an *in vivo* infection model to assess the impact of *HP0858* disruption on *H. pylori* virulence by evaluating bacterial viability within the larval hemocoel. Unlike mammalian hosts, *G. mellonella* larvae have a protective cuticle analogous to mammalian skin, serving as a physical barrier. When the cuticle is breached, the larvae activate their humoral innate immune response, producing soluble defense compounds such as antimicrobial peptides, lytic enzymes, and opsonins. Simultaneously, the cellular innate immune system is stimulated, enhancing the larvae’s ability to combat invading pathogens [53]. The proliferation of hemocytes triggers cellular immune responses, including phagocytosis, nodulation, and melanogenesis, providing a robust defense mechanism [23,54]. In general, hemocytes in the hemocoel engulf invading microorganisms, similar to the role of mammalian neutrophils, and release respiratory burst products, such as free radicals and peroxides. These compounds contribute to tissue damage, mirroring the immune response seen in humans [55]. Additionally, melanogenesis, evidenced by observable color changes in infected larvae, reflects the immune system’s response to bacterial presence in the gut or body cavity. This process is thought to protect endogenous tissues from damage caused by pathogen elimination. Our findings demonstrated that *H. pylori* virulence in larvae is both time- and dose-dependent. Notably, the *HP0858*-disrupted mutant exhibited significantly reduced virulence compared to the WT strain 26,695 and the *HP0858* complemented strain (Figure 7(b)). Overall, these results suggest that *G. mellonella* larvae provide a valuable *in vivo* model for investigating the role of LPS biosynthesis in *H. pylori* virulence.

## Conclusion

We demonstrated that the enzyme encoded by the *HP0858* gene plays a critical role in heptose biosynthesis. Disruption of this gene in *H. pylori* results in a truncated LPS structure, lacking the complete O-antigen and much of the core region. The resulting mutant exhibited hypersensitivity to the detergent SDS and the antibiotic novobiocin, increased surface hydrophobicity, and a tendency to autoaggregate. Additionally, the *HP0858* knockout mutant showed a significant reduction in its ability to adhere to and internalize gastric AGS cells. Infected AGS cells also exhibited a diminished hummingbird phenotype and a marked decrease in IL-8 secretion. Furthermore, the mutant’s reduced virulence was confirmed using the *Galleria mellonella* wax moth as an in vivo *H. pylori* infection model. The pivotal role of RfaE/HldE/HP0858 in bacterial fitness and virulence makes it a promising target for the future development of novel preventive and therapeutic strategies against *H. pylori* infections.

## Supplementary Material

Supplementary Fig 2_2.jpg

HP0858_paper_Supplementary_material_20241230 (1).docx

Supplementary Fig 1_2.jpeg

## Data Availability

The data supporting this study’s findings are openly available in Figshare at https://doi.org/10.6084/m9.figshare.28081937
