# Supplementary figures and images for: Roles of the lipopolysaccharide biosynthesis-related gene HP0858 in the fitness of Helicobacter pylori and its virulence in Galleria mellonella
Source: Virulence. 2025 Aug 24;16(1):2548620. doi: 10.1080/21505594.2025.2548620 (PMC12377144; doi:10.1080/21505594.2025.2548620)

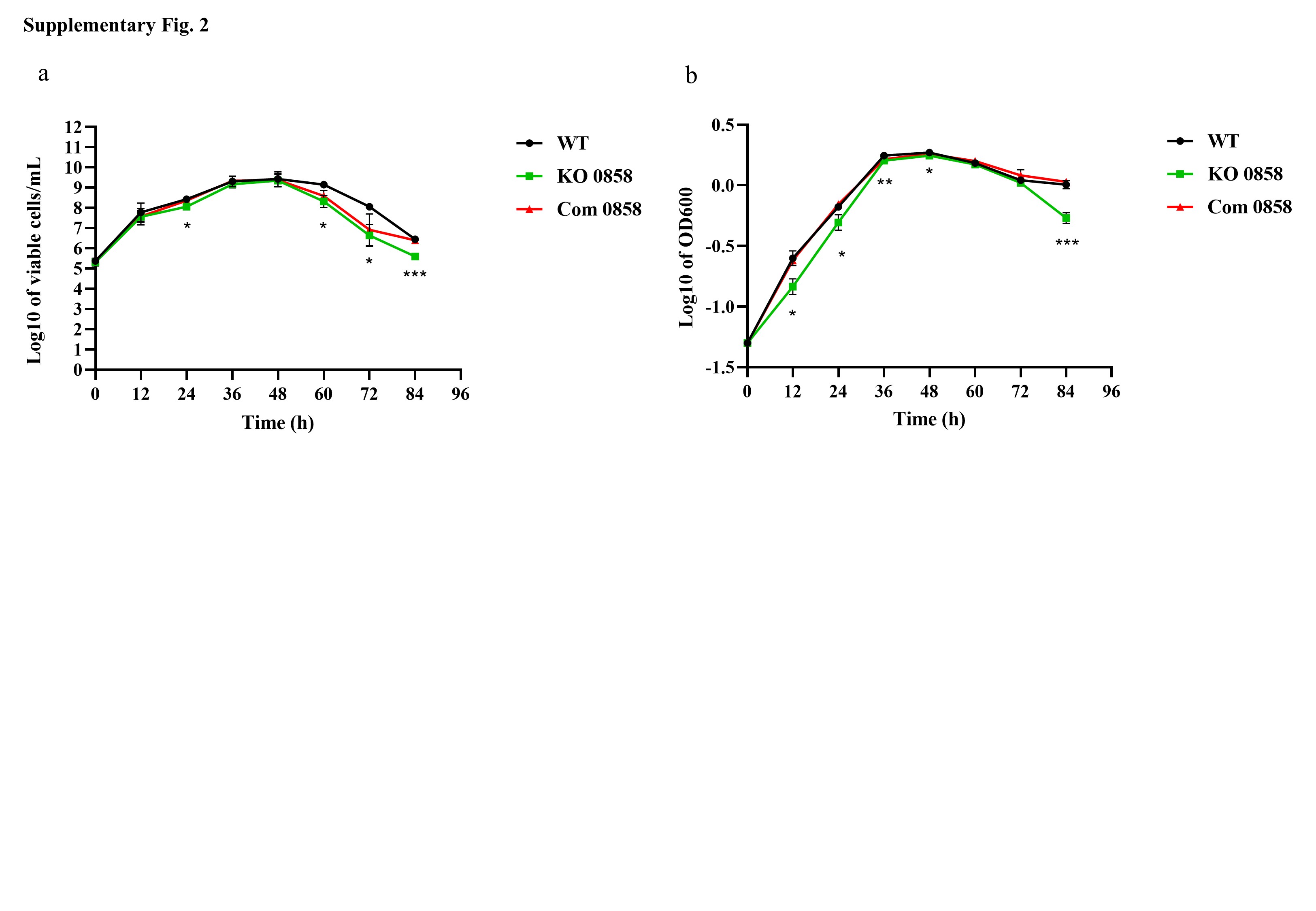

Supplement: Supplementary Fig 2_2.jpg [file KVIR_A_2548620_SM6948.jpg]

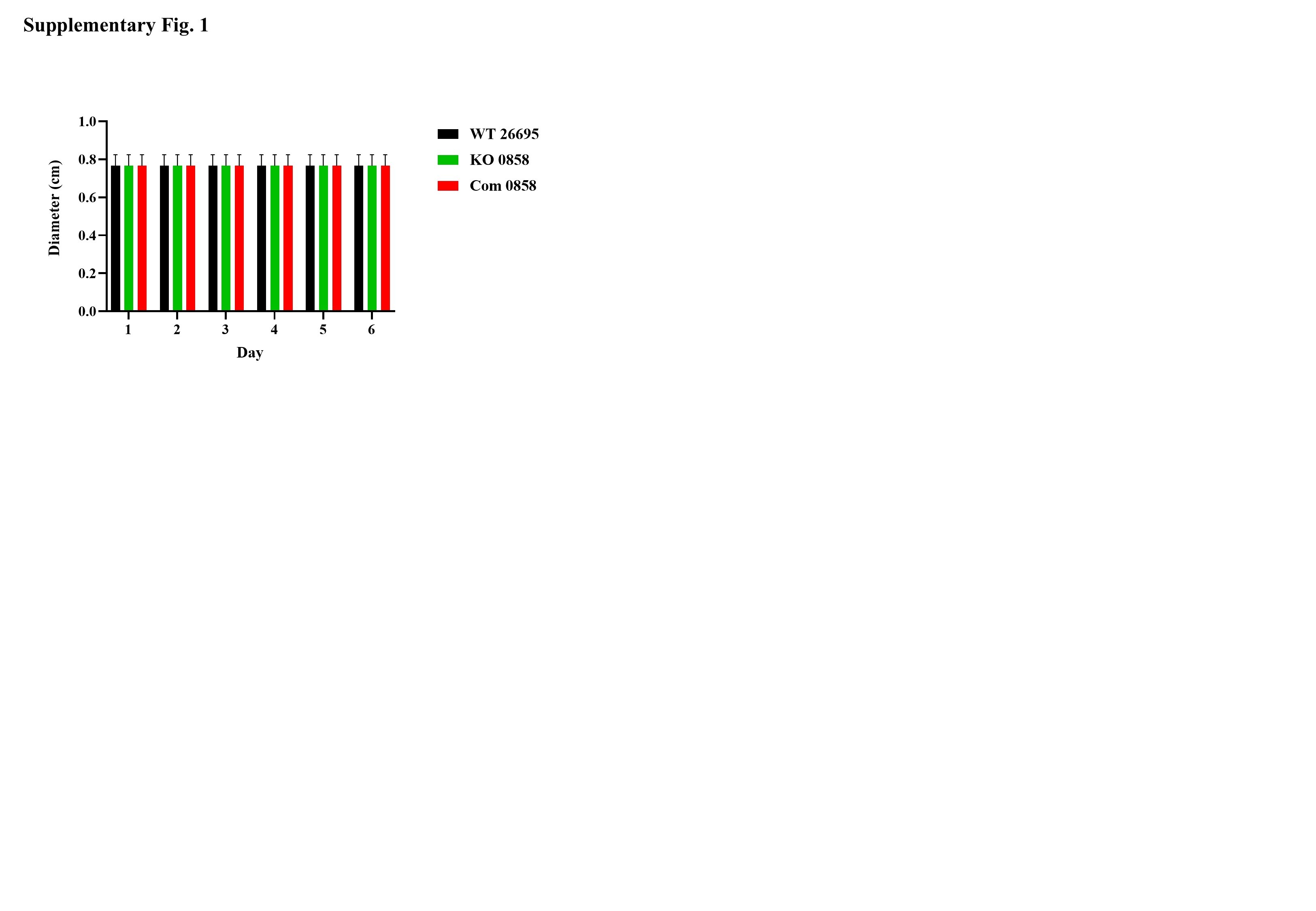

Supplement: Supplementary Fig 1_2.jpeg [file KVIR_A_2548620_SM6946.jpeg]
