# Supplementary material for: Roles of the lipopolysaccharide biosynthesis-related gene HP0858 in the fitness of Helicobacter pylori and its virulence in Galleria mellonella
Source: Virulence. 2025 Aug 24;16(1):2548620. doi: 10.1080/21505594.2025.2548620 (PMC12377144; doi:10.1080/21505594.2025.2548620)
Supplement: HP0858_paper_Supplementary_material_20241230 (1).docx [file KVIR_A_2548620_SM6947.docx]

**Supplementary materials**

**Supplementary Figure 1.** The motility of the *H. pylori* strains used in this study. 10 µL of the bacterial culture **(**OD_600_ =1.0**)** were inoculated onto soft agar plates containing 0.5% agar. The diameter of the bacterial growth halo (in centimeters, cm) was monitored and measured over a 6-day period. The data presented reflect the average halo diameter calculated from three independent trials. WT, wild-type strain; KO, knockout mutant; Com, complemented strain.

a

b

**Supplementary Figure 2.** Re-evaluation of the bacterial growth curves of the *H. pylori* 26695 strain WT and its derived strains by using the plate counting method alongside the OD_600_ measurements. Bacterial growth was analyzed using the plate counting technique (a) and spectrophotometric measurements of OD_600_ values (b) at various time intervals. The resulting growth curves are presented as semi-logarithmic graphs, with the y-axis showing either the average CFU counts (a) or the average OD_600_ readings (b) on a logarithmic scale, while the x-axis represents time on a linear scale. The data were obtained from triplicate tests with statistical analysis (unpaired, two-tailed Student’s t-test; * p < 0.01, ** p < 0.01, *** p < 0.001). WT, wild-type strain; KO, knockout mutant; Com, complemented strain.
